# Enhancing FDM Rapid Prototyping for Industry 4.0 Applications Through Simulation and Optimization Techniques

**DOI:** 10.3390/ma18194555

**Published:** 2025-09-30

**Authors:** Mihalache Ghinea, Alex Cosmin Niculescu, Bogdan Dragos Rosca

**Affiliations:** Department of Robots and Manufacturing Systems, Faculty of Industrial Engineering and Robotics, National University of Science and Technology Politehnica Bucharest, 060042 Bucharest, Romania or niculescualexcosmin@gmail.com (A.C.N.); or bogdan.rosca.drago@gmail.com (B.D.R.)

**Keywords:** rapid prototyping, additive manufacturing, Klipper firmware, process optimization

## Abstract

Modern manufacturing is increasingly shaped by the paradigm of Industry 4.0 (Smart Manufacturing). As one of its nine pillars, additive manufacturing plays a crucial role, enabling high-quality final products with improved profitability in minimal time. Advances in this field have facilitated the emergence of diverse technologies—such as Fused Deposition Modelling (FDM), Stereolithography (SLA), and Selective Laser Sintering (SLS)—allowing the use of metallic, polymeric, and composite materials. Within this context, Klipper v.0.12, an open-source firmware for 3D printers, addresses the performance limitations of conventional consumer-grade systems. By offloading computationally intensive tasks to an external single-board computer (e.g., Raspberry Pi), Klipper enhances speed, precision, and flexibility while reducing prototyping time. The purpose of this study is twofold: first, to identify and analyze bottlenecks in low-cost 3D printers and second, to evaluate how these shortcomings can be mitigated through the integration of supplementary hardware and software (Klipper firmware, Raspberry Pi, additional sensors, and the Mainsail interface). The scientific contribution of this study lies in demonstrating that a consumer-grade FDM 3D printer can be significantly upgraded through this integration and systematic calibration, achieving up to a 50% reduction in printing time while maintaining dimensional accuracy and improving surface quality.

## 1. Introduction

Additive manufacturing techniques have been developed to meet the increasing demand for producing complex geometries with minimal material waste. The advancement of rapid prototyping has driven progress in this field, including the reduction of printing defects and the improvement of the mechanical performance of printed parts. Among these techniques, Fused Deposition Modelling (FDM) has emerged as the most widely adopted approach, particularly through the use of thermoplastic polymer filaments [1,2]. The FDM process involves heating a continuous filament and extruding layer by layer to fabricate three-dimensional objects. Its main advantages are cost efficiency, short processing times, and ease of operation. Nevertheless, FDM exhibits several limitations, such as restricted mechanical strength, visible layer lines, surface roughness, and a limited range of compatible thermoplastic materials [3,4].

To overcome these limitations, new firmware solutions have been developed. Klipper, an open-source firmware for 3D printers, integrates processors from single-board computers with printer mainboards, thus expanding the computational capacity of additive manufacturing systems. In conventional systems, firmware typically relies on a single microcontroller to process G-code files and execute machine instructions. As printers incorporate increasingly complex features, these microcontrollers often struggle to manage the associated computational workload. Klipper addresses this constraint by distributing computational tasks across multiple processors, thereby enabling faster response times, improved precision, and enhanced scalability [5].

In addition to Klipper, this study further employed Mainsail, a web-based user interface that enables remote monitoring and control of 3D printers [6]. Through Mainsail, users can adjust machine parameters, initiate print jobs, and track progress from any device with a web browser. This integration enhances usability, workflow flexibility, and accessibility, thereby facilitating the management of 3D printing operations in both research and industrial environments. When combined, Klipper and Mainsail offer a comprehensive framework that enhances performance while aligning with the broader objectives of Industry 4.0 and Smart Manufacturing ecosystems.

### 1.1. Principles of FDM Printing

Fused Deposition Modelling (FDM) represents the most widely used technique in additive manufacturing today [1]. The process starts from a digital three-dimensional model, which serves as input alongside a spool of thermoplastic filament. Inside the printer’s hot end, the filament is heated to temperatures typically ranging from 180 °C to 260 °C, depending on the selected material, surpassing its glass transition temperature and melting point.

Once molten, the polymer is extruded through a nozzle with diameters commonly ranging from 0.2 mm to 0.8 mm. On the build platform, the material is deposited layer by layer, with individual layers generally between 0.1 mm and 0.3 mm thick. As each layer cools and solidifies, the structure is progressively formed, ultimately resulting in the complete three-dimensional object [3,7,8].

### 1.2. Materials and Process Parameters

FDM printers employ a diverse selection of thermoplastic filaments, each exhibiting distinct mechanical and thermal characteristics:

- Polylactic Acid (PLA): A biodegradable thermoplastic polymer that is easy to process, characterised by a relatively low melting point (180–220 °C) and a glass transition temperature in the range of 60–65 °C.
- Acrylonitrile Butadiene Styrene (ABS): A thermoplastic polymer exhibiting higher mechanical strength and thermal resistance compared to PLA. Its melting point ranges from 220 to 260 °C, with a glass transition temperature of approximately 105 °C. The material is, however, more challenging to process.
- Polyethylene Terephthalate Glycol (PETG): A copolymer providing a favorable balance of mechanical strength, ductility, and thermal stability, with a melting point of 220–250 °C and a glass transition temperature around 80 °C [9,10].

#### The Print Head and Printer Resolution

The print head represents a crucial element of the Fused Deposition Modelling (FDM) process, incorporating the extrusion nozzle. Nozzles are typically constructed from brass, owing to its superior thermal conductivity, or from hardened steel, which enhances wear resistance when processing abrasive filaments [11,12]. Fundamental printing parameters, such as nozzle diameter, layer height, extrusion width, and the positional accuracy of the printer’s kinematics, directly determine the achievable resolution and dimensional fidelity of the fabricated components [13].

### 1.3. Objectives of the Study

The present study aims to enhance the overall performance and output quality of a specific 3D printer through systematic observation and experimental evaluation, leveraging the integrated capabilities of the Klipper firmware. The study addresses two principal objectives:

(1) Improvement of the surface quality of printed components,
(2) Enhancement of printing speeds without compromising dimensional accuracy or structural integrity.

The influence of Klipper’s firmware functionalities on multiple dimensions of 3D printing—including surface quality, print reliability, and process efficiency—is evaluated, with quantification of the achievable improvements under practical conditions. Analysis of the experimental outcomes provides empirical evidence of the extent to which Klipper’s embedded tools contribute to both surface quality enhancement and reduced printing times, offering insights for optimized additive manufacturing workflows.

The methodology begins with an iterative calibration procedure that employs a 20 × 20 × 20 mm calibration cube as a reference model for systematic performance diagnostics. Deviations or printing defects identified during this phase are mitigated through firmware-assisted correction tools, and the calibration cube is subsequently reprinted to evaluate the effectiveness of these adjustments. This iterative process frequently reveals secondary challenges not detected during initial printing, enabling continuous optimization of printing accuracy, surface quality, and overall system reliability.

For both the quality and performance improvement studies, PLA filaments sourced from multiple manufacturers were employed. Following resolution of all identifiable part quality issues through iterative problem-solving, a series of experiments was conducted to increase print speed, assessing their effects on actual print times in conjunction with elevated acceleration settings. This approach facilitates a comprehensive evaluation of material-dependent performance, kinematic optimization, and overall throughput, providing a detailed understanding of the interplay between print quality and process efficiency.

The novelty of this study lies in the proposal and validation of an integrated methodology for enhancing the performance of consumer-grade FDM printers through the combination of supplementary hardware and software with advanced calibration procedures. Unlike previous works, which usually address isolated aspects of 3D printer optimisation, our approach demonstrates a reproducible pathway to simultaneously increase printing speed while maintaining dimensional accuracy and improving surface quality. The contribution of this paper is therefore both methodological and applicative, offering an accessible solution for bridging low-cost additive manufacturing with the requirements of Industry 4.0 and Small and Medium Enterprises (SME) environments.

## 2. Experimental Setup

In this section, we present the experimental setup employed in this study, including the hardware configuration of the 3D printer, the implemented mechanical modifications, and the firmware/software integration. This dedicated description ensures clarity and reproducibility of the experimental workflow.

### 2.1. Hardware Setup

#### 2.1.1. Test Platform: Tevo Black Widow Printer

**Figure 1:**
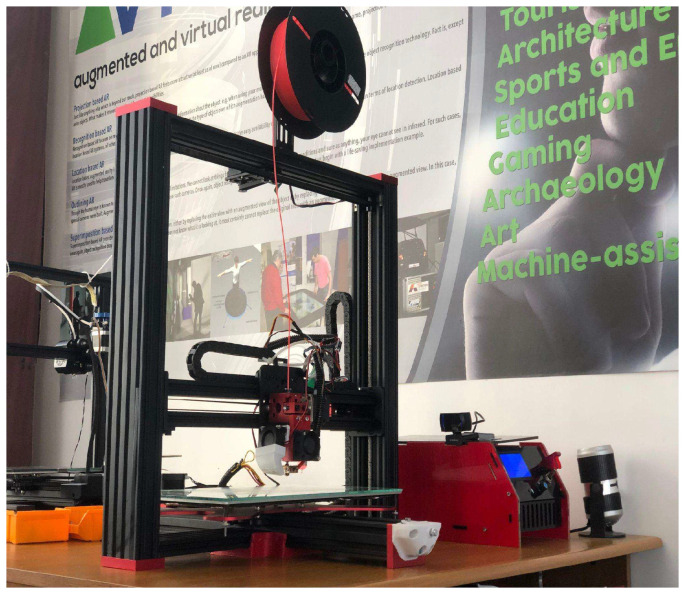
Experimental setup: Tevo Black Widow 3D printer (produced by TEVO Printers, Zhanjiang, China) with hardware modifications and Klipper firmware v.0.12 integration.

The Tevo Black Widow represents a DIY 3D printer kit with a build volume of 370 mm × 250 mm × 300 mm [14]. It is equipped with a metal direct-drive extruder featuring a 5:1 gear ratio to enhance available torque and support 1.75 mm filament. The system further comprises a 400 × 250 mm heated bed, an MKS MOSFET-based heating controller, anodized CNC-milled plates to improve structural rigidity, and an ANTCLABS BLTouch probe for bed levelling (Figure 1).

#### 2.1.2. Mechanical Modifications

The initial assembly of the 3D printer was systematically inspected to ensure precise alignment and secure fastening of all components, with lubrication applied to the Z-axis lead screws to minimize friction and enhance motion stability.

Thermal management—cooling shroud: A custom cooling shroud was fabricated and installed to direct and concentrate airflow through the hotend fins, thereby improving heat dissipation, reducing thermal gradients, and mitigating heat creep, which can negatively affect printing accuracy.

Part cooling optimization: Due to the absence of integrated part cooling in the original kit, a blower-style fan with a simple air duct was installed to augment convective cooling of the deposited layers. This intervention effectively addressed a critical bottleneck in print speed and surface quality.

Mechanical precision—belt tensioning mechanism: A screw-based tensioner for the Y-axis belt was printed and mounted, enabling precise and reproducible tension adjustments, thereby enhancing dimensional accuracy and overall system reliability.

Build plate flatness optimization—glass bed: The initial heated bed exhibited concavity; a borosilicate glass plate was subsequently adopted to provide a flat, thermally stable, and easily cleanable surface. This modification facilitated uniform first-layer deposition, improved part adhesion, and simplified part removal after cooling, contributing to enhanced print consistency and reliability.

### 2.2. Software/Firmwaare Setup

The printer’s operation is controlled via an MKS Gen v1.4 board executing Marlin firmware [15]. The open-source Klipper firmware was employed due to its advanced capabilities, including simplified and efficient printer tuning and configuration, Wi-Fi connectivity, and remote access. These features allow modern software-driven control while offloading computationally intensive tasks to an external Raspberry Pi. The configured printer setup is illustrated in Figure 1.

The above configuration represents the complete experimental setup used throughout our investigation, forming the basis for the calibration, optimization, and performance evaluation described in the following sections.

## 3. Experimental Methodology

In order to ensure a transparent and reproducible investigation, this section presents the experimental methodology adopted in the study. The methodology is structured to clearly distinguish between constant process parameters, which were kept unchanged across all experiments, and variable process parameters, which were systematically modified to evaluate their influence on printing performance.

**Figure 2:**
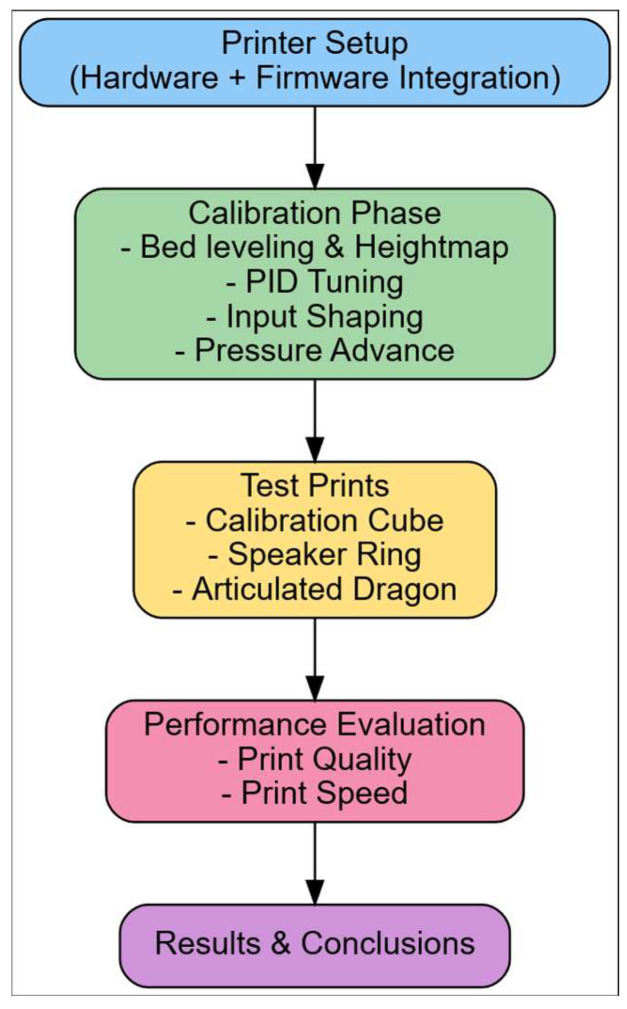
Workflow of the experimental methodology, from printer setup and calibration to performance evaluation.

The overall methodology of this study is summarized in Figure 2. The flowchart outlines the main stages, from hardware and firmware setup through calibration (bed levelling, PID tuning, input shaping, and pressure advance) to benchmark test prints and final performance evaluation. This structured representation ensures clarity and reproducibility of the experimental procedure.

### 3.1. Constant Process Parameters

The constant parameters used throughout the study are listed in Table 1. These values were fixed for all experimental runs in order to provide a stable baseline for comparison.

Table 1 and Table 2 summarize the experimental settings, listing the constant parameters (e.g., nozzle diameter, filament type, layer height, bed and nozzle temperatures) and the variable parameters (e.g., print speed, acceleration, input shaping, PID tuning, part cooling, and pressure advance). These tables provide a concise overview of the testing conditions and the range of values considered.

### 3.2. Variable Process Parameters

The variable parameters were systematically adjusted to evaluate their effect on printing performance. The tested values are summarized in Table 2.

### 3.3. Methodological Workflow

In Figure 2, the overall workflow of the investigation is illustrated. It depicts the sequential steps from printer setup and calibration to parameter tuning, test printing, and final performance evaluation. This framework ensures that each stage of the research is logically connected and that the effects of individual optimizations can be systematically assessed.

### 3.4. Definition of Parameter Ranges

The levels and ranges of the process variable parameters were defined based on a combination of manufacturer specifications, commonly reported values in the literature, and preliminary test prints. For example, the print speed values (40–100 mm/s) were selected to cover the typical operating range of the Tevo Black Widow printer, (produced by TEVO Printers, Zhanjiang, China) while including higher values to evaluate performance under stress conditions. The acceleration levels (500–4000 mm/s^2^) correspond to the limits allowed by the Klipper firmware and the mechanical capabilities of the printer. The PID tuning parameters were adjusted according to the recommended self-tuning routines in Klipper, while the pressure advance values (0.0–0.1) were chosen based on ranges reported in previous studies and validated by test prints. The part cooling levels (0%, 50%, 100%) reflect practical extremes and a setting in the middle. These ranges ensured that the experiments covered both realistic printing conditions and relevant limit scenarios for performance optimization.

### 3.5. Statistical Consistency of Experimental Runs

To ensure reproducibility and reliability of the obtained results, each experimental condition was repeated three times under identical settings. This approach minimizes the impact of random fluctuations arising from machine dynamics, filament inhomogeneity, or environmental conditions, which are known to influence FDM performance.

The reported values in the Results section correspond to the mean of the repeated trials. Variability is expressed as mean ± standard deviation (SD), which represents the dispersion of the measured values around the average. This statistical measure provides a transparent indication of consistency and allows the reader to assess the robustness of the findings.

Since the primary aim of this study was to demonstrate the impact of firmware-driven optimizations on process efficiency, advanced statistical models (e.g., ANOVA or Taguchi designs) were not applied. Instead, the adopted approach provides an appropriate balance between methodological rigor and clarity, highlighting the significance of the observed improvements while avoiding unnecessary complexity.

This experimental methodology provides a structured framework that combines clearly defined process parameters with a systematic calibration and evaluation workflow. By distinguishing between constant and variable parameters and by following a stepwise approach from hardware setup to performance testing, the study ensures both repeatability and transparency. The methodology thus establishes a solid foundation for analysing the effects of firmware-driven optimization on FDM printing performance, as presented in the subsequent sections.

## 4. Process Calibration and Optimization

### 4.1. Bed Flatness Evaluation (Heighmap)

**Figure 3:**
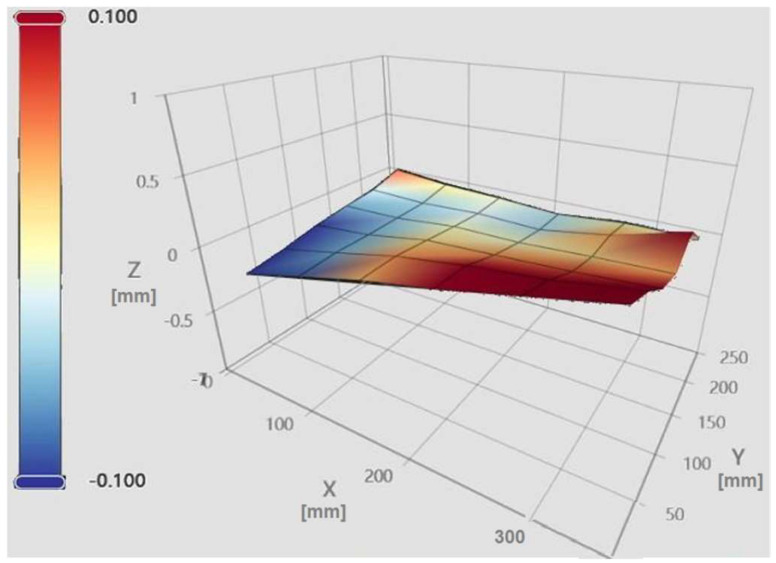
The mesh of our initial print bed.

**Figure 4:**
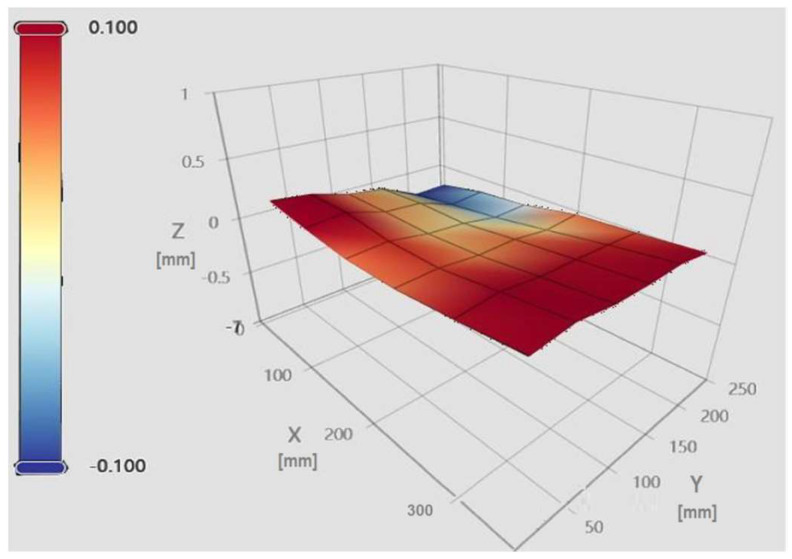
Bed mesh of the glass bed.

The “Heightmap” functionality enables systematic probing of multiple points across the print bed, which are subsequently visualized as a mesh. The Klipper firmware records the maximum and minimum elevations, providing an estimate of the peak-to-peak variation measured by the bed probe. In this study, a 6 × 6 grid of evenly spaced points was employed to generate the mesh [15]. Figure 3 and Figure 4 illustrate the Mainsail web interface in the “Heightmap” panel, from which bed mesh calibration can be initiated, the printer can be homed, and previously saved meshes can be loaded for visualization [6].

Each bed mesh is associated with a unique identifier (NAME), a grid size (6 × 6), maximum and minimum values relative to the origin (centre of the print bed), and the corresponding range between these extremes [16]. The panel additionally allows toggling the flat reference plane, the wireframe, and the calculated mesh, providing a comprehensive view of bed topography and height deviations relative to the origin.

As shown in Figure 3, the stock heated bed exhibits a concave surface with a maximum elevation of +0.364 mm and a minimum of −0.214 mm, yielding a peak-to-peak variation of 0.578 mm. The color gradient illustrates the relative height variations, with red indicating elevated regions and blue indicating lower regions.

In contrast, the glass bed mesh, presented in Figure 4, demonstrates substantially improved planarity. Quantitative measurements confirm a maximum height of +0.179 mm, a minimum of −0.129 mm, and a reduced peak-to-peak variation of 0.308 mm. These results indicate a significantly flatter and more uniform bed surface, contributing to enhanced print stability and dimensional fidelity. The color gradient (red = elevated regions, blue = lower regions) illustrates surface height variations across the build area.

### 4.2. Proportional–Integral–Derivative (PID) Tuning for Thermal Stability

A calibration cube was initially printed to assess the printer’s performance, revealing inconsistencies in layer height. Mechanical inspection confirmed that the Z-axis lead screws were functioning properly, indicating that the observed irregularities were not due to mechanical faults. Further analysis identified fluctuating nozzle and bed temperatures as the primary factor limiting print quality. To address this issue, the Proporțional–Integral–Derivativ (PID) tuning functionality in Klipper was employed, effectively stabilizing the thermal environment and improving layer height uniformity. This intervention resulted in enhanced print reliability and improved dimensional accuracy [17,18].

The Proportional–Integral–Derivative (PID) controller is a widely adopted control strategy in both industrial and scientific applications. In this study, it is utilized as a digital temperature regulation system, ensuring optimized dynamic response and steady-state accuracy. Operating within a closed-loop feedback architecture, the controller continuously evaluates the deviation between the setpoint and the measured process variable. The proportional component provides immediate correction proportional to the error magnitude, the integral component eliminates residual steady-state error by integrating the cumulative deviations over time, and the derivative component anticipates future trends by assessing the rate of change in the error. The combined action of these three terms minimizes steady-state error, improves transient response, and enhances overall robustness of temperature control in additive manufacturing processes [19,20,21].

**Figure 5:**
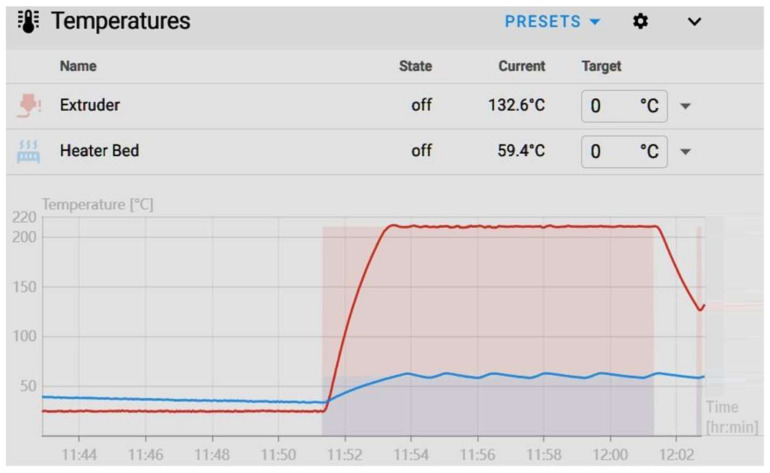
Bed temperature fluctuations (blue line).

Figure 5 illustrates a setpoint tracking error, in which the measured temperature exhibits oscillatory deviations around the reference value. The observed fluctuations above and below the target indicate inadequate thermal stability and reveal limitations in the control system’s capacity to maintain steady-state accuracy (red line is the extruder temperature).

To calibrate the thermal control system, the console commands:

PID_CALIBRATE HEATER = heater_bed TARGET = 60

and

SET_HEATER_TEMPERATURE HEATER = extruder TARGET = 210

were executed, corresponding to the nominal printing conditions of 60 °C for the heated bed and 210 °C for the extruder. Upon execution, the firmware initiates a thermal response characterization routine, during which multiple heating and cooling cycles are conducted. This procedure allows for the determination of the proportional, integral, and derivative parameters for each heating element. The resulting PID coefficients are subsequently applied to regulate the heaters, thereby minimizing oscillatory deviations, enhancing setpoint tracking accuracy, and ensuring steady-state thermal stability throughout the printing process [22].

After storing the optimized PID coefficients using the printer’s configuration commands, the system’s thermal stability was verified, confirming that the target temperatures were successfully maintained. To quantitatively evaluate the impact of the tuning on print quality, a calibration cube was subsequently printed under the updated parameters.

**Figure 6:**
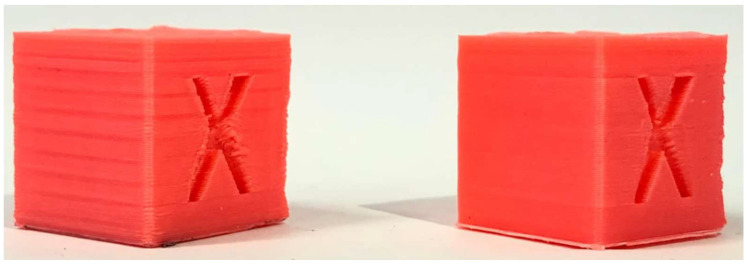
Cube before (**left**) and after PID tuning (**right**).

The PID optimization effectively eliminated the previously observed horizontal banding on the cube (Figure 6, right). However, this adjustment also made another defect more prominent. This phenomenon, commonly referred to as ringing or ghosting, appears as repetitive horizontal patterns along the printed layers [23]. Such quality issues are induced by mechanical vibrations within the printer structure, which high printing speeds, elevated acceleration, or positional deviations in the motion system can exacerbate. These artefacts reflect the printer’s dynamic response to rapid directional changes and highlight the interplay between control parameters and the mechanical characteristics of the printing platform.

### 4.3. Input Shaping for Vibration Mitigation

As previously discussed, the Klipper firmware integrates advanced control strategies designed to mitigate vibrations and oscillatory dynamics inherent to 3D printing systems. A central element of this approach is the use of input shaping, commonly referred to as motion shapers. These algorithms modify the printer’s motion commands to counteract mechanical resonance and reduce the amplitude of vibrations generated during rapid accelerations or directional changes [24,25,26]. Over time, several types of motion shapers have been developed, including Zero Vibration (ZV), Modified Zero Vibration (MZV), and Extra Insensitive (EI) input shapers, each characterized by distinct capabilities in attenuating specific vibrational modes. By implementing these techniques, Klipper enhances positional accuracy, minimizes layer ringing, and improves overall print quality, particularly when operating at elevated printing speeds [24,25,26,27]. These are:

1. ZV (Zero Vibration) represents the simplest form of input shaping, consisting of two equally spaced impulses. This shaper is specifically designed to suppress a single dominant resonance frequency while introducing minimal temporal delay into the motion system. Although it provides fast execution and low latency, its vibration attenuation capabilities are limited, and its performance is highly sensitive to inaccuracies in the estimated resonance frequency. Consequently, while ZV is suitable for fast-response applications, precise modelling of the system dynamics is essential to achieve optimal results.
2. MZV (Modified Zero Vibration) is an extension of the ZV shaper, achieved by introducing a third impulse into the shaping sequence. This enhancement increases the shaper’s robustness to modelling inaccuracies and provides improved attenuation of residual vibrations compared to the basic ZV approach. By distributing the impulses more effectively, MZV shows a higher tolerance to small errors in resonance frequency estimation, reducing sensitivity to parameter uncertainties. However, this increased robustness comes at the cost of a slightly longer execution delay. As a result, MZV offers a favorable balance between vibration suppression performance and latency, making it well-suited for general-purpose motion control applications. Nevertheless, its performance may still degrade under conditions of significant frequency drift.
3. EI (Extra Insensitive) is designed to be less sensitive to inaccuracies in resonance frequency detection. It offers a better balance between vibration suppression and robustness compared to ZV or MZV shapers. EI is moderately complex, with a longer impulse duration, providing enhanced resilience to resonance drift. While it introduces a slightly higher delay, it delivers reliable overall performance, making it particularly suitable for systems with variable or uncertain resonance characteristics.
4. 2HUMP_EI (Two-Hump Extra Insensitive) extends EI by using additional impulses, allowing better suppression of both primary and harmonic frequencies. It is highly robust and reduces ghosting in prints, but introduces a notable execution delay. 2HUMP_EI provides excellent vibration suppression and improved print surface quality, though it responds more slowly. This shaper is ideal for printers with strong structural resonance and visible ghosting.
5. 3HUMP_EI (Three-Hump Extra Insensitive) is the most advanced member of the EI family. It employs additional impulses to flatten vibrations over a wider frequency range, achieving excellent ghosting suppression. This comes at the cost of the highest execution delay. 3HUMP_EI offers the best overall vibration reduction and extremely smooth prints, but its high latency may limit printing speed or require careful acceleration tuning. It is particularly suitable for high-speed printers or those with CoreXY kinematics with high precision requirements.

Table 3 presents the main characteristics of Klipper input shapers for 3D printer vibration compensation, focusing on low-speed operation or cases requiring fast response. Data are derived from official documentation, source code, scientific literature, and community experience [28,29,30,31].

Input shaping is a control feature implemented in the Klipper firmware. It represents an open-loop technique that generates a command signal specifically designed to cancel mechanical vibrations of the 3D printer. A highly accurate method for tuning shaper parameters involves direct measurement of printer resonances using an accelerometer [32].

In this study, an MPU6050 gyroscope-accelerometer module was interfaced with a Raspberry Pi to perform resonance measurements. Two tests were conducted using the Klipper TEST_RESONANCES command: one for the X-axis and one for the Y-axis. Each test produced CSV files containing vibration data, which were subsequently processed and visualised using a custom script executed on the Raspberry Pi.

**Figure 7:**
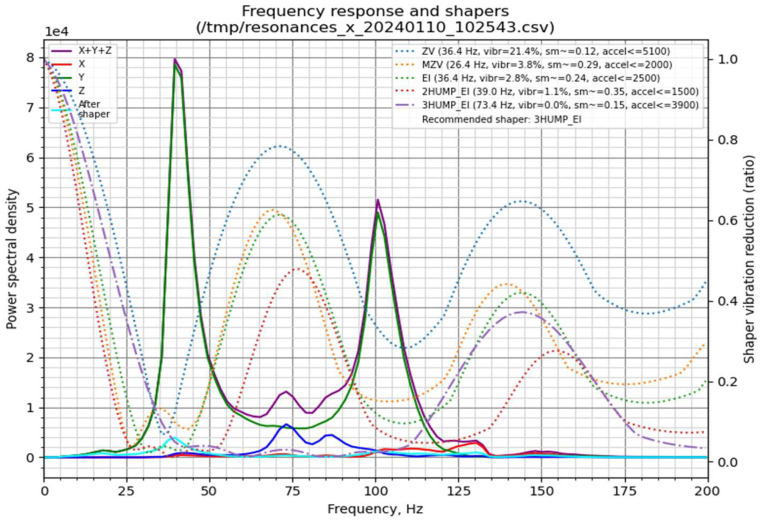
Graph generated by the stand-alone script for the X-axis.

**Figure 8:**
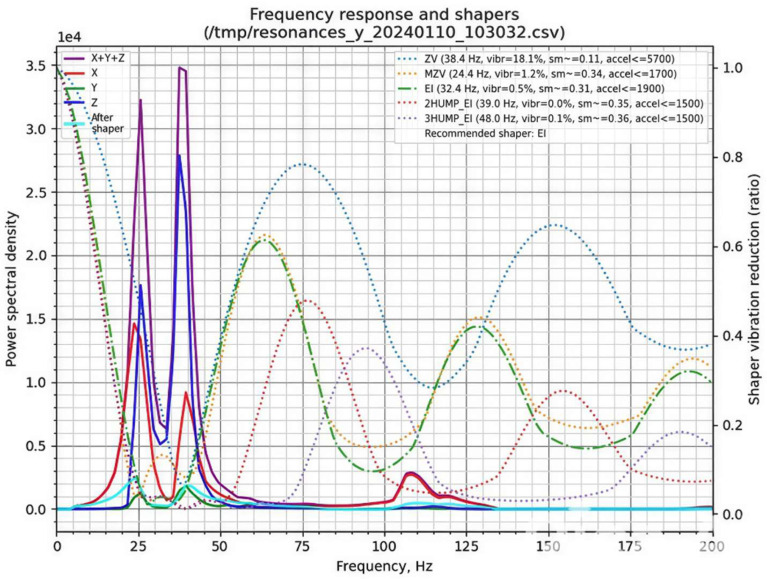
Graph generated by the stand-alone script for the Y-axis.

The resulting frequency response graphs (Figure 7 and Figure 8) illustrate the effectiveness of different input shapers in mitigating mechanical vibrations along the X (Figure 7) and Y (Figure 8) axes. These graphs provide a direct quantification of vibration suppression achieved through Klipper’s Input Shaping functionality.

In the graphs, the left Y-axis corresponds to the power spectral density (PSD), representing vibration energy, with higher values indicating stronger vibrations. The colored curves denote the spectral distribution along the X, Y, and Z axes, whereas the thick black curve labelled “After shaper” shows the residual vibrations following the application of a shaper. The right Y-axis represents the shaper vibration reduction ratio, quantifying the efficiency of each filter in suppressing vibrations at specific frequencies. The X-axis corresponds to frequency (Hz), indicating the natural resonances of the printer. Peaks in the spectral plots correspond to frequencies at which vibrations are most pronounced.

Conceptually, these resonances can be likened to a mass-spring system, where the printhead or print bed is supported by belts exhibiting elastic behavior. In an ideal, perfectly rigid printer frame, the frequency response would exhibit a single peak corresponding to the mass on its respective belt. In our measurements, both axes display a primary peak representing the resonance frequency of the belt-driven mass. A secondary peak at approximately 40 Hz appears in both axes, likely associated with frame resonance due to structural compliance. Additional smaller peaks are observed at 70–75 Hz for the X-axis and 105–110 Hz for the Y-axis. These minor peaks may result from suboptimal belt tension, loose fasteners, axis friction, or imperfect mounting of the gyroscope during testing.

The presented graphs (Figure 7 and Figure 8) provide extensive information on the vibration characteristics of the 3D printer axes. Of particular relevance (top-right section) are the Klipper input shapers—ZV, MZV, 2HUMP_EI, 3HUMP_EI, and EI—represented by their respective dotted lines. Additionally, the plots indicate the recommended shaper for maximal vibration attenuation, the “sm” parameter (smooth time), and the “accel” parameter (maximum recommended acceleration for the corresponding axis). Based on the analysis of both X- and Y-axis responses, a compromise between vibration suppression and printing performance was selected.

For belt-driven (bed-slinger) printers, it is generally recommended to adopt the lowest of the two axis-specific recommended accelerations. In the present study, this corresponds to the Y-axis employing the EI shaper at an acceleration of 1900 mm/s^2^. However, to achieve an optimal balance between printing speed and part quality, the acceleration was increased to 2500 mm/s^2^.

Additionally, it is important to consider the complementary data obtained from the gyroscope measurements. In the top-left region of the graphs, the resonance response of each axis (X, Y, and Z) is displayed, with the purple line representing the combined response of all three axes, and the cyan line illustrating the expected frequency response of the system after applying the recommended input shaper.

**Figure 9:**
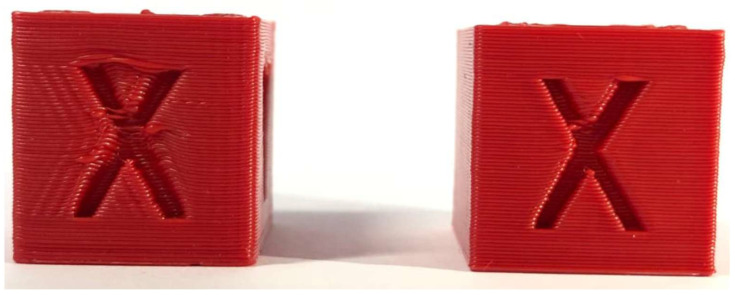
Cube without Input Shaper (**left**), cube with Input Shaper (**right**).

The selected acceleration of 2500 mm/s^2^ substantially exceeds the stock value of 1000 mm/s^2^. Nonetheless, increasing acceleration alone does not produce a proportional reduction in print time. The implementation of the Input Shaper enables the use of higher print speeds within the slicer while mitigating the ghosting effect, thereby preserving print quality (Figure 9).

### 4.4. Pressure Advance Calibration

The Pressure Advance feature mitigates the occurrence of oozing and improves extrusion accuracy. Ideally, during an extrusion move, a consistent volume of filament should be deposited along the entire trajectory. However, using standard extrusion algorithms often results in under-extrusion at the start of a move and over-extrusion at the end [33].

**Figure 10:**
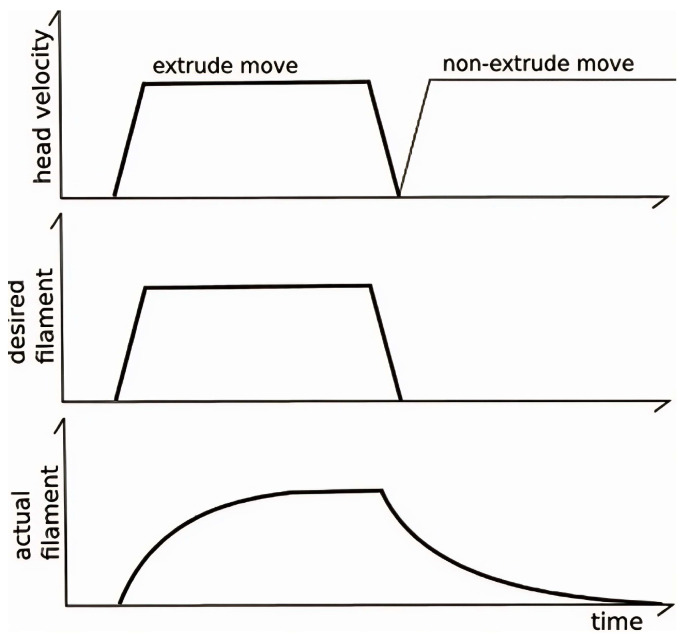
Extruder moves following [33].

Pressure Advance overcomes this limitation by employing a dynamic model of the extruder that accounts for the relationship between filament, internal pressure, and flow rate. Unlike traditional models that assume instantaneous filament exit, Klipper models extrusion based on pressure propagation within the system, as illustrated in Figure 10.

The corrected extruder position is calculated as:

(1) $$p_{a\_p}=p_{n}+K\cdot v_{n}$$

where

- *p_a_p_* (pa_position) is the adjusted extruder position,
- *p_n_* (nominal_position) is the planned position without compensation,
- *v_n_* (nominal_velocity) is the planned filament feed velocity,
- *K* is the pressure_advance_coefficient, which scales the correction based on the filament dynamics.

Direct application of this formula may induce abrupt velocity changes in the extruder motor. To mitigate these effects, Klipper incorporates a “smoothing” algorithm, ensuring stable extrusion while maintaining accurate filament deposition and consistent print quality [34].

**Figure 11:**
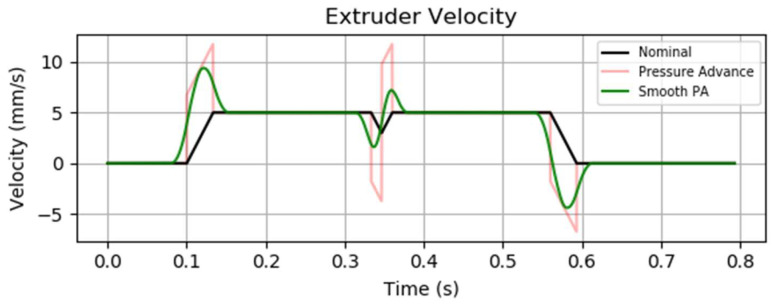
Extruder velocity over time PA graph from the Klipper3D website [33].

The graph presented in Figure 11 illustrates an example of two extrusion moves connected by a non-zero cornering velocity. The pressure advance system actively compensates for extrusion lag by introducing additional filament into the extruder during acceleration phases [7]. The magnitude of this compensation is directly proportional to the desired flow rate, meaning that higher flow rates require more filament to be injected in order to maintain pressure equilibrium. Conversely, during deceleration, the surplus filament is retracted, resulting in a negative velocity of the extruder motor.

Calibration of the pressure advance parameter requires a fully operational printer, as the tuning process involves the fabrication and visual inspection of a dedicated test object. For this purpose, a G-code file was generated using a slicing program to print a large hollow square with no infill, a layer height set to 75% of the nozzle diameter (0.4 mm nozzle), and a print speed of 100 mm/s. The calibration was conducted using the following commands:

Calibration of the pressure advance parameter requires a fully operational printer, as the tuning process involves the fabrication and visual inspection of a dedicated test object. For this purpose, a G-code file was generated using a slicing program to print a large hollow cube with no infill, a layer height set to 75% of the nozzle diameter (0.4 mm nozzle), and a print speed of 100 mm/s. The calibration was conducted using the following commands:

SET_VELOCITY_LIMIT SQUARE_CORNER_VELOCITY = 1 ACCEL = 500

and

TUNING_TOWER COMMAND = SET_PRESSURE_ADVANCE PARAMETER = ADVANCE START = 0 FACTOR = 0.005

and started the print.

**Figure 12:**
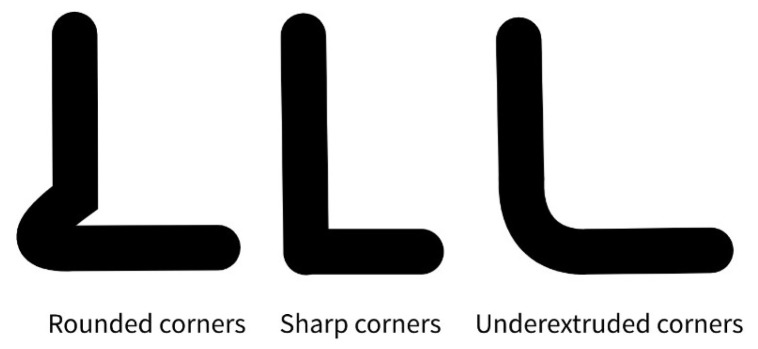
Representation of the change in the corner shape.

The ‘TUNING_TOWER’ command instructs Klipper to incrementally modify the pressure_advance (PA) parameter across successive layers of the printed object. Consequently, higher layers correspond to larger pressure_advance values, while the ‘SET_VELOCITY_LIMIT’ command enforces reduced cornering speeds, thereby accentuating the influence of extruder pressure. As illustrated in Figure 12, this methodology produces a progressive variation in corner sharpness: at low pressure_advance values, excess material deposition results in rounded corners; at intermediate values, optimal compensation yields sharp and well-defined corners; whereas at excessively high pressure_advance values, overcompensation leads to underextrusion and visibly deficient corners [7,8].

**Figure 13:**
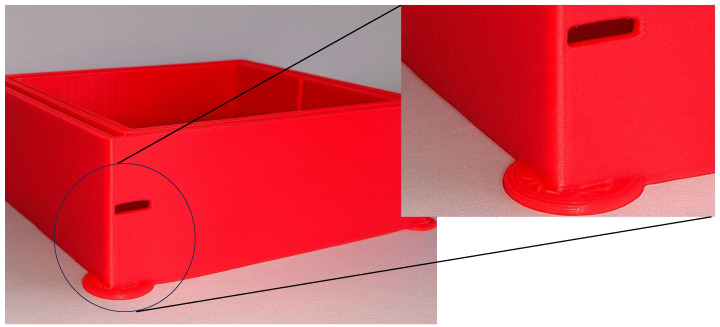
Pressure Advance calibration tower, indicating the optimal compensation value at 13.5 mm height.

After approximately 30 min of printing, we observed that the region of the tower corresponding to the sharpest corners had already been reached, while at higher layers clear signs of under-extrusion began to appear; therefore, the print was stopped (Figure 13). The optimal corner was measured at a height of approximately 13.50 mm from the base and was marked accordingly. Applying the calibration formula for the pressure advance parameter,

(2) $$P_{A}=P_{A\;start}+h_{m}\cdot k$$

where

- *P_A_* is the final value of the *pressure advance* parameter,
- *P_A start_* is the initial set value of pressure advance,
- *h*_*m*_ is the measured height on the calibration tower [mm],
- *k* is the calibration factor specific to the configuration [mm^−1^].

In our case: *P_A_* = 0 + 13.50 × 0.005 = 0.0675, which was subsequently saved into the printer configuration file.

## 5. Performance Improvements

### 5.1. Theoretical Performance Limits

The maximum attainable print speed is constrained by two principal factors: (i) the vo lumetric extrusion capacity of the nozzle and (ii) the efficiency of the subsequent cooling process. Since the cooling fan already operates at full capacity during the experiments, further improvements in cooling efficiency would necessitate alternative strategies, such as employing a higher-capacity fan, implementing multiple fans, or optimizing the cooling duct geometry.

To evaluate the extrusion capability of the system, we conducted test extrusions of 20 mm of filament at different feed rates. The results indicated that the hotend can sustain a volumetric flow rate of approximately 12 mm^3^/s at an extrusion feed rate of 5 mm/s when using the installed 0.4 mm nozzle. The volumetric flow rate is defined as:

*Q* = *v* · *w* · *h* (3)

where

*Q* is the volumetric flow rate [mm^3^/s],
*v* is the print speed [mm/s],
*w* is the line width [mm], and
*h* is the layer height [mm] [35,36].

By applying this relation, the theoretical maximum print speed of the current configuration is estimated to be 150 mm/s, for a nozzle diameter of 0.4 mm and a layer height of 0.2 mm.

### 5.2. Benchmark Models and Experimental Results

To assess the practical implications of the implemented calibrations and optimization, we conducted a series of comparative print tests. Three representative models were selected, designed to capture different geometric and functional aspects of additive manufacturing:

- A calibration cube—a simple, compact geometry with limited features, particularly suited for assessing dimensional accuracy and surface quality [37].
- A speaker ring—a circular geometry that requires simultaneous movement of both X- and Y-axes, making it useful for evaluating dynamic performance during continuous curvature printing [12].
- An articulated dragon—a complex, organic model with multiple joints, chosen to evaluate the system’s capability to handle high accelerations, variable printi.ng speeds, and the likelihood of error propagation in intricate geometries [38].

Slicing operations were performed using Ultimaker Cura 5.5.0, one of the most widely adopted slicing engines in the 3D printing community [8]. The Tevo Black Widow printer was configured as the reference profile, with baseline prints executed using Cura’s default Draft (0.3 mm layer height) and Normal (0.2 mm layer height) profiles, without further modifications.

For the test prints, Creality CR-PLA Fluo-Red filament was used, processed at a nozzle temperature of 210 °C and a heated bed temperature of 60 °C. These conditions were kept constant across all trials to ensure comparability.

In the following subsections, we present a detailed description of the printing procedures, including modifications applied to slicer parameters, and provide a quantitative analysis of the improvements in build time and surface quality.

#### 5.2.1. Calibration Cube

To establish a reference for speed-related performance improvements, a standard 20 × 20 × 20 mm calibration cube was fabricated under two different velocity configurations [37]. For the fast print, we used the Normal 0.2 mm profile with a 0.4 mm nozzle, a print speed of 100 mm/s, no generated support structures, brim build plate adhesion, and ironing enabled.

The slow print was performed under identical slicing conditions, except for the print speed, which was reduced to 50 mm/s. The fast configuration completed in 23 min 2 s, with an average of 13.9 s per layer, while the slow configuration required 30 min 38 s, with an average of 18.5 s per layer. This outcome corresponds to an approximate 33% reduction in print time.

**Figure 14:**
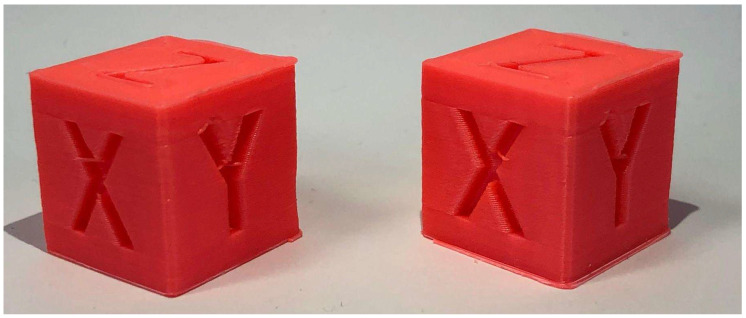
Calibration Cube 50 mm/s (**left**) next to Calibration Cube 100 mm/s (**right**).

Figure 14 presents a visual comparison of the calibration cube obtained at 50 mm/s (left) and at 100 mm/s (right).

#### 5.2.2. Speaker Ring

The second benchmark model selected was a speaker ring, chosen due to its circular geometry that simultaneously engages both the X- and Y-axes, thereby offering a suitable case for assessing speed-related artifacts [12]. For the fast print, the slicing was performed with the Normal 0.2 mm profile and a 0.4 mm nozzle, at a print speed of 100 mm/s. Supports were generated, build plate adhesion was ensured via a brim, the infill structure was set to gyroid, and ironing was enabled.

The slow print was produced under identical conditions, except that the print speed was reduced to 50 mm/s. The fast configuration completed in 1 h 49 min 33 s, with an average of 1 min 46 s per layer, compared to the slow configuration, which required 3 h 17 min 57 s, with an average of 3 min 15 s per layer. This corresponds to an approximate 55% reduction in printing time.

**Figure 15:**
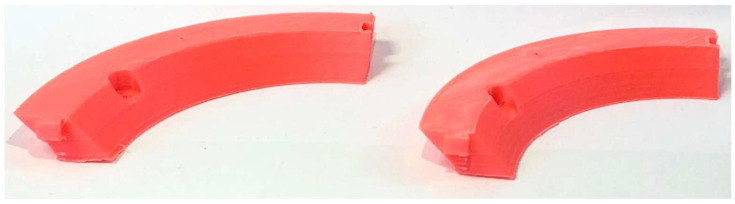
Speaker Ring 50 mm/s (**left**) next to Speaker Ring 100 mm/s (**right**).

Figure 15 illustrates the comparison between the speaker ring fabricated at 50 mm/s (left) and at 100 mm/s (right).

#### 5.2.3. A Complex 3D Model (Articulated Dragon)

The third benchmark model was an articulated dragon [38], selected to test the printer under more complex and organic geometries, where higher accelerations and increased printing speeds could provide significant advantages. For the fast print, slicing was carried out using the Normal 0.2 mm profile with a 0.4 mm nozzle, at a print speed of 100 mm/s. Supports were generated, and build plate adhesion was achieved with a brim.

The slow print was produced under identical conditions, except with the print speed reduced to 50 mm/s. The fast configuration completed in 7 h 54 min 13 s, with an average of 3 min 47 s per layer, compared to the slow configuration, which required 12 h 15 min 2 s, with an average of 5 min 52 s per layer. This represents an approximate 55% reduction in printing time.

**Figure 16:**
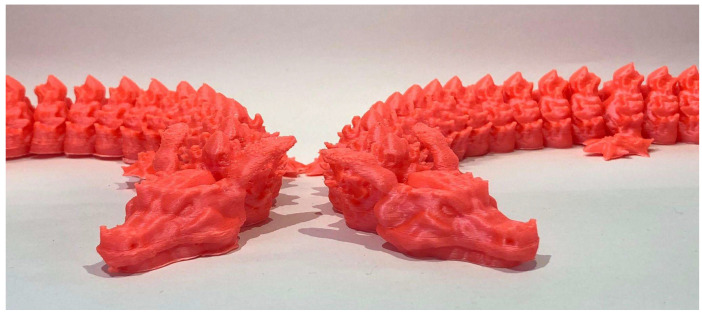
Articulated dragon 50 mm/s (**left**) next to articulated dragon 100 mm/s (**right**).

The optimized printing parameters yielded consistent time reductions across all test models: approximately 33% for the Calibration Cube and 55% for both the Speaker Ring and the Articulated Dragon. These results confirm that the applied calibrations significantly enhance printing efficiency without compromising print quality. Figure 16 illustrates the comparison between the 3D complex model fabricated at 50 mm/s (left) and at 100 mm/s (right).

## 6. Discussion

### 6.1. Limitations of Our Study

**Figure 17:**
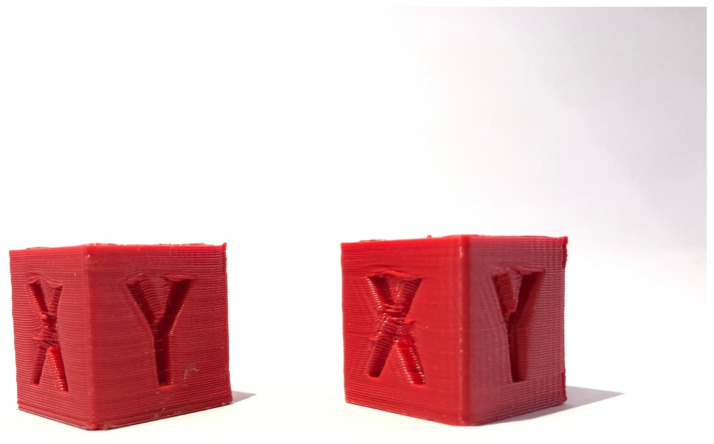
Calibration cube printed 50 mm/s before all improvements (**left**), next to calibration cube printed at 100 mm/s after all improvements are applied.

The experimental results demonstrated that it is possible to simultaneously improve surface finish and reduce printing time, achieving an average time reduction of 47.6%. The quality of the prints improved markedly, transitioning from surfaces with multiple defects to parts exhibiting only minor imperfections. These improvements, combined with the reduction in print duration to nearly half, confirm that the intended objective—enhancing print quality without sacrificing efficiency—was successfully achieved, as illustrated in Figure 17.

Nevertheless, certain limitations were observed. In particular, the Calibration Cube showed only a 33% reduction in print time compared to the more substantial gains achieved with the other two geometries. This discrepancy is most likely attributable to the slicer parameter minimum layer time, which enforces a lower bound on the duration of each layer to prevent overheating, deformation, or warping.

Such constraints are especially relevant for models with small cross-sectional areas, where individual layers are completed very quickly. Further improvements could be obtained by enhancing the part cooling system, thereby allowing for the adjustment of the minimum layer time parameter to lower values without compromising print integrity.

### 6.2. Future Research Directions

In our study, several constraints should be acknowledged. First, only a 0.4 mm nozzle was employed, without testing alternative nozzle sizes or geometries that could influence print speed and accuracy. Second, the experiments were limited to PLA, thus excluding other commonly used polymers such as PETG or ABS, which may exhibit distinct thermal and rheological behaviors. Furthermore, PLA from a single manufacturer was used, although material properties across suppliers can vary and potentially affect both achievable print speed and surface quality. In terms of hardware, the testing was restricted to a Cartesian “bed-slinger” printer, without considering alternative architectures such as CoreXY or Delta, which could respond differently to the implemented optimizations. Similarly, no alternative slicer configurations or strategies were investigated, and the hotend used was not compared with other designs that may exhibit different melting and extrusion characteristics.

Taken together, these limitations indicate that while the observed improvements in print quality and efficiency are robust within the tested setup, the generalizability of the findings remains conditioned by specific printer configurations, materials, and process parameters. It is therefore expected that the relative benefits of techniques such as Input Shaper or Pressure Advance would vary across different system setups and material selections.

A consolidated overview of the experimental outcomes is provided in Table 4. The data confirm that, across all benchmark models, the implemented optimizations led to consistent reductions in print time—up to 55%—while maintaining dimensional accuracy within ±0.30 mm and achieving visible improvements in surface quality. This synthesis reinforces the robustness of the proposed methodology and sets the stage for the concluding remarks presented in the next section.

## 7. Conclusions

This study addressed the limitations of a consumer-grade FDM 3D printer by implementing the Klipper firmware together with systematic calibration techniques, including PID tuning, input shaping, and pressure advance. The optimizations enabled a doubling of the nominal print speed from 50 mm/s to 100 mm/s while maintaining dimensional accuracy and significantly improving surface finish.

Experimental validation across three benchmark models confirmed these findings. The calibration cube demonstrated dimensional fidelity at 20 × 20 × 20 mm; the speaker rings fitted precisely without geometric distortion, and the articulated dragon preserved complex geometrical details without visible surface degradation. Quantitatively, print time reductions reached up to 55%, while average improvements across models were 47.6%, demonstrating that higher productivity can be achieved without compromising print quality.

These results underscore the value of Klipper as an open-source firmware capable of extending the performance of low-cost 3D printers. By reducing production times while sustaining surface and dimensional quality, Klipper aligns with the broader objectives of Industry 4.0, where additive manufacturing is a key technological pillar. Its integration with enabling technologies such as the Industrial Internet of Things (IIoT), simulation, and artificial intelligence will further support the transition to Industry 5.0, enabling more adaptive, sustainable, and high-precision manufacturing systems. Future integration with AI-driven slicing and digital twins may further enhance FDM performance.

Limitations and Constraints: This study was limited to a single 3D printer platform (Tevo Black Widow) and a specific set of firmware-driven optimizations. The experiments were performed under controlled laboratory conditions, with the PLA filament as the only tested material. As such, direct generalization of the results to other machines, materials, or industrial environments should be made with caution. In addition, advanced statistical optimization methods (e.g., Taguchi designs or ANOVA) were not applied, as the primary objective was to demonstrate the feasibility and impact of the implemented firmware-based improvements.

Future Work: Future research will extend the methodology to multiple printer platforms, different materials, and varying environmental conditions. The use of advanced statistical designs for multi-parameter optimization, long-term reliability studies, and the integration of additional firmware features will be considered. Moreover, the application of machine learning-based tuning and digital twin frameworks offers promising directions to further enhance process adaptability and performance.

## Figures and Tables

**Table 1:** Constant experimental parameters used in the study.

Parameter	Value	Notes
Nozzle diameter	0.4 mm	Brass, standard E3D-type
Filament type	PLA, 1.75 mm	Same spool used for all tests
Layer height	0.2 mm	Fixed for all experiments
Bed temperature	60 °C	Maintained constant
Nozzle temperature	200 °C	Maintained constant
Infill density	20%	Rectilinear pattern
Ambient temperature	23 ± 2 °C	Laboratory conditions

**Table 2:** Variable process parameters and tested levels.

Parameter	Levels Tested	Description
Print speed	40, 60, 80, 100 mm/s	Standard G-code speed
Acceleration	500, 1000, 2000, 4000 mm/s^2^	Firmware settings
Input shaping	Enabled/Disabled	Implemented in Klipper
PID tuning	Default/Tuned	Auto-tuning of hotend and bed
Part cooling fan	0%, 50%, 100%	Adjusted via firmware
Pressure advance	0.0/0.05/0.1	Klipper linear advance

**Table 3:** Comparative Table of Input Shapers.

Shaper	Vibration Reduction	Print Quality (Ghosting)	Execution Delay	Notes
ZV	Basic	Medium	Low	Simplest form, fast but limited
MZV	Good	Better than ZV	Slightly more	Compromise between reduction and delay
EI	Robust	Good	Moderate	Insensitive to resonance drift
2HUMP_EI	Very good	Low ghosting	Higher	Better smoothing of high-frequency noise
3HUMP_EI	Excellent	Minimal ghosting	Highest	Best for high-speed printers with complex resonance

**Table 4:** Summary of benchmark test results before and after optimization.

Benchmark Model	Print Speed (mm/s)	Print Time (h:mim:s)	Time Reduction (%)	Dimensional Accuracy (mm Deviation)	Surface Quality
Calibration Cube	50 → 100	0:30:38 → 0:23:02	~33%	±0.20	Improved (minor defects)
Speaker Ring	50 → 100	3:17:57 → 1:49:33	~55%	±0.25	Smooth, reduced artifacts
Articulated Dragon	50 → 100	12:15:02 → 7:54:13	~55%	±0.30	Preserved fine details

## Data Availability

The original contributions presented in this study are included in the article. Further inquiries can be directed to the corresponding author.
